# Laryngoscope and a New Tracheal Tube Assist Lightwand Intubation in Difficult Airways due to Unstable Cervical Spine

**DOI:** 10.1371/journal.pone.0120231

**Published:** 2015-03-24

**Authors:** Cai-neng Wu, Wu-hua Ma, Jian-qi Wei, Hua-feng Wei, Qing-yun Cen, Qing-xiang Cai, Ying Cao

**Affiliations:** 1 Department of Anesthesiology, the First Affiliated Hospital of Guangzhou University of Traditional Chinese Medicine, Guangzhou, China; 2 Department of Anesthesiology and Critical Care, University of Pennsylvania, Philadelphia, Pennsylvania, United States of America; 3 School of Pharmacological Science, Southern Medical University, Guangzhou, China; Erasmus Medical Centre, NETHERLANDS

## Abstract

**Purpose:**

The WEI Jet Endotracheal Tube (WEI JET) is a new tracheal tube that facilitates both oxygenation and ventilation during the process of intubation and assists tracheal intubation in patients with difficult airway. We evaluated the effectiveness and usefulness of the WEI JET in combination with lightwand under direct laryngoscopy in difficult tracheal intubation due to unstable cervical spine.

**Methods:**

Ninety patients with unstable cervical spine disorders (ASA I-III) with general anaesthesia were included and randomly assigned to three groups, based on the device used for intubation: lightwand only, lightwand under direct laryngoscopy, lightwand with WEI JET under direct laryngoscopy.

**Results:**

No statistically significant differences were detected among three groups with respect to demographic characteristics and C/L grade. There were statistically significant differences between three groups for overall intubation success rate (p = 0.015) and first attempt success rate (p = 0.000). The intubation time was significantly longer in the WEI group (110.8±18.3 s) than in the LW group (63.3±27.5 s, p = 0.000) and DL group (66.7±29.4 s, p = 0.000), but the lowest SpO2 in WEI group was significantly higher than other two groups (p<0.01). The WEI JET significantly reduced successful tracheal intubation attempts compared to the LW group (p = 0.043). The severity of sore throat was similar in three groups (p = 0.185).

**Conclusions:**

The combined use of WEI JET under direct laryngoscopy helps to assist tracheal intubation and improves oxygenation during intubation in patients with difficult airway secondary to unstable spine disorders.

**Trial Registration:**

Chinese Clinical Trial Registry ChiCTR-TRC-14005141

## Introduction

Patients with cervical spine instability usually present with limited range of neck motion and a challenged airway for tracheal intubation. The operators (more and more emergency department and ICU doctors do the intubation) for tracheal intubation must consider two potentially competing factors, securing the airway while minimizing neck motion. Manual in-line stabilization (MILS) is recommended during direct laryngoscopy and intubation in patients with known or suspected cervical spine instability [1]. However, MILS often worsens direct laryngeal visualization and therefore raises possibility of difficult tracheal intubation [2].

The lightwand is a simple technique that has been proven to be safe and effective in difficult intubation [3–5]. It is usually difficult to intubate patients with unstable cervical spines by direct laryngoscopy. It was recommended the using of the lightwand in a presumed unstable cervical spine injury over the Macintosh laryngoscope [6].

A special tracheal tube designed by Huafeng Wei named WEI Jet Endotracheal Tube (WEI JET; Wei Medical LLC, Cherry Hill, NJ) applies supraglottic jet oxygenation and ventilation (SJOV) during tracheal intubation and assists tracheal intubation blindly in patients with difficult airway [7].We hypothesized that combined use of lightwand and WEI JET under direct laryngoscopy will maintain oxygenation/ventilation during tracheal intubation and assist tracheal intubation in patients with difficult airway due to unstable cervical spine. Thus, we compared the effectiveness and complication of tracheal intubation using regular endotracheal tube and lightwand only, regular endotracheal tube with lightwand under direct laryngoscope, and WEI JET with lightwand under direct laryngoscopy.

## Materials and Methods

### Ethics Statement

This study was approved by the First Affiliated Hospital of Guangzhou University of Traditional Chinese Medicine. Written informed consent was obtained from all patients.

### Patients and Study Design

Ninety patients with unstable cervical spine disorders (ASA I-III) under general anaesthesia were enrolled from July 2014 to September 2014. Patients with the following types of condition were excluded: age <18 years; had risk of gastric aspiration; increased intracranial pressure; obesity (BMI> 35); relevant drug allergy; abnormalities of the upper airway, polyps, tumors, abscesses, inflammation; or had foreign bodies in the upper airway. The protocol for this trial and supporting CONSORT checklist are available as supporting information; see S1 CONSORT Checklist and S1 Protocol.

Patients recruited in this study were randomly assigned by computer-generated randomization schedule. There groups were the LW group (intubations were performed only by lightwand and regular endotracheal tube), the DL group (lightwand used in conjunction with regular endotracheal tube under direct laryngoscopy), and the WEI group (lightwand used in conjunction with WEI JET under direct laryngoscopy).

Each patient was placed in supine position with a neutral head position. MILS was created with a cervical immobilization collar (Stifneck Select, Leardal Medical GmbH, Germany). The appropriate size of MILS was determined by finger-sizing method following the manufacturer’s instructions. This device effectively downsizes mouth opening and limits neck extension. Patients were given 5 min of pre-oxygenation before anesthesia induction. Non-invasive blood pressure, SpO_2_, ECG were monitored and recorded every minute. Anaesthesia was induced with propofol target control infusion (4μg.ml^−1^) and remifentanil (3.5ng.ml^−1^) followed by succinylcholine (1mg.kg^−1^) [8]. After anesthesia induction, direct laryngoscopy was performed with a Macintosh size 3 blade, the trachea was intubated by one of four anesthesiologists, who was experienced with jet ventilation and had completed at least 50 successful tracheal intubations using lightwand before the study. The glottis view was described by Cormack and Lehane (C/L) and divided into four grades (1 = full view of the glottis; 2 = partial view of the glottis; 3 = only the epiglottis visible; and 4 = neither epiglottis nor glottis visible.), without applying external laryngeal pressure. More than two attempts required would be defined as a failure intubation. Then, the airway was managed by following the anesthesiologist’s former experience.

In the LW group, tracheal intubations were performed only by using lightwand in which introduced a regular tracheal tube (7-mm ID tube in women, and an 7.5-mm ID tube in men). The tip of the tube/lightwand combination was bent to a 90° angle [9]. Room lights were dimmed, and the combination was introduced into the oral cavity and repositioned in the midline until its entry into the oropharynx. When the tip of lightwand was placed inside the glottis, a well-defined circumscribed glow could be seen in the anterior neck [10]. If esophageal intubation happened, the transmitted glow was diffused and the procedure was tried again. After removed the lightwand, proper tracheal tube placement was confirmed by end-tidal carbon dioxide monitoring.

In the DL group, tracheal intubation was performed with lightwand in combination with regular endotracheal tube under direct laryngoscopy. A Macintosh size 3 blade was used for lightwand intubations. The blade was held in the non-dominant hand before insertion of the tracheal tube-lightwand combination. Tracheal intubation was performed under direct vision of the glottis (C/L Grade 1 or 2). For patients with Grade 3 glottis view, the lightwand was passed underneath the epiglottis. For Grade 4, tracheal intubation was accomplished with midline technique [11–12]. When an optimal transillumination was obtained, tracheal intubation was achieved. If transillumination was not seen in larynx, the procedure was tried again.

**Fig 1:**
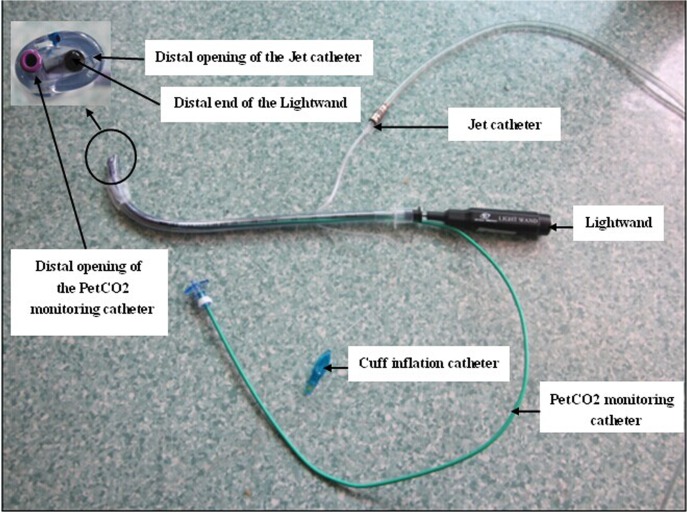
Illustration of components and assembly of the WEI JET in combination with lightwand.

In the WEI group, the WEI JET took the place of regular tracheal tube which combined with the direct laryngoscopy and lightwand. As shown in **Fig. 1**, The WEI JET contained two additional parts compared to a regular endotracheal tube: a catheter for end-tidal CO_2_ pressure (PetCO_2_) monitoring and a jet catheter for ventilation. The jet catheter with an ID 2.0 mm was built into the anterior wall of WEI JET. Jet ventilation was performed using a manual jet ventilator (Manujet III, VBM Medizintechnik GmbH, Germany) was connected to the proximal end of jet catheter. The operator adjusted the distal tip of the lightwand until the midline illumination was observed in the anterior neck, the maximum stable PetCO_2_ and chest rise were achieved [7–8]. The WEI JET was then slid into the trachea over the lightwand and the PetCO_2_ monitoring catheter.

Awake fiberoptic intubation has been recommended for unanticipated difficult airway [13]. Although experienced anesthesiologists make the procedure seem simple, fiberoptic intubation is a difficult technique to learn and master, and if rarely used, competence is difficult to maintain [14]. In addition, it requires patient cooperation and ready-to-use devices, which cannot be achieved sometime. This technique requires more time than direct laryngoscopy, even when performed by an expert [15]. Thus, alternative management techniques should be available.

The primary endpoints were the rate of successful intubation and the time taken to intubate. The intubation time was defined as from insertion of the intubation device between the teeth to the first breath of controlled mechanical ventilation and detection of positive PetCO_2_. The secondary endpoints were the number of intubation attempts and the lowest SpO_2_ during intubation. After the procedure, patients were asked about the severity of sore throat using a VAS (0 = no pain, 10 = worst imaginable pain) at discharge from the post-anesthesia recovery room.

### Statistical Analysis

The sample size was calculated based on the hypothesis that a combination of WEI JET, lightwand and direct laryngoscope will increase intubation successful rate during the intubation. The sample size was also calculated based on a pilot study of 30 patients.We calculated that the overall intubation success rate was 80% for lightwand, 90% for direct laryngoscopy in combination with lightwand and 100% for the WEI JET. Based on these figures, accepting a two-tailed α error of 5% and β error of 20%, we calculated that 27 patients would be required for each group. Assuming the potential for patients to drop out of the study, the total sample size was increased to 90 patients.

All data were reported as mean ± SD, absolute number (n), or percentages. Statistical analyses were performed with the Statistical Package for Social Sciences Software (SPSS 18.0 for windows; SPSS Inc., Chicago, IL, USA). The intubation time, lowest SpO_2_ during intubation, VAS score and number of intubation attempts were analysed using ANOVA for three group comparisons. Bonferroni correction was performed between-group comparisons, as appropriate. Kruskal-Wallis was used to analyse C/L grade. Fisher’s exact test was used to analyse rate of successful intubation. A *P*-value < 0.05 was considered to be statistically significant.

## Results

A total of 90 patients were recruited into the study. No statistically significant difference was detected between groups in demographic characteristics, glottis view grade under direct laryngoscopy (**Table 1**). Patients were not conditioned with significant respiratory disease which might contribute to SpO_2_ baseline.

**Table 1:** Characteristics of Patients in Group LW, Group DL and Group WEI.

Characteristic	Group LW	Group DL	Group WEI	P value
Patients,n	24	29	30	-
Male, n (%)	8(33.3)	9(31.0)	9(30.0)	0.460
Age (yrs)	42.3±16.2	37.9±12.1	40.9±11.1	0.268
Weight (kg)	58.5±10.4	61.8±8.7	60.6±6.9	0.224
Height (cm)	162.0±7.0	164.8±7.9	163.7±6.7	0.268
BMI (kg/m^2^)	22.2±3.1	22.7±2.8	22.6±2.2	0.526
Mouth opening (cm)	2.8±0.33	3.0±0.37	2.9±0.33	0.450
C/L Grade, n (%)				0.551
1	0(0)	0(0)	0(0)	-
2	3(12.5)	3(10.3)	2(6.7)	-
3	21(87.5)	26(89.7)	27(90)	-
4	0(0)	0(0)	1(3.3)	-

Patients in WEI group were successfully intubated on the first attempt. In contrast, five patients needed second attempt in the LW group and four in the DL group. Patients in all groups were successfully intubated except for six patients who were excluded due to intubation failure in the LW group and one in the DL group. There were statistically significant differences between three groups for overall intubation success rate (*p* = 0.015) and first attempt success rate (*p* = 0.000). With regard to the overall intubation success rate and first attempt success rate, there were significant difference between LW group and WEI group (*p* = 0.024, *p* = 0.000, respectively)(**Table 2**).

**Table 2:** Comparison of Monitored Values in Group LW, Group DL and Group WEI.

Parameter	Group LW	Group DL	Group WEI	p(overall)
	n = 24	n = 29	n = 30	
Overall intubation success rate, n (%)	24(80)^a^	29(96.7)^b^	30(100)*	0.015
Success at first attempt, n (%)	19(63.3)^a^	25(83.3)^b^	30(100)**	0.000
Intubation time (s)	63.3±27.5	66.7±29.4	110.8±18.3** ^††^	0.000
Number of insertion attempts	1.21±0.4	1.14±0.4	1.0±0*	0.041
Lowest SpO_2_ during intubation (%)	96.6±2.8	98.4±2.1**	100±0** ^††^	0.000
Sore throat,VAS score	2.0±0.8	2.3±0.7	2.4±0.6	0.185

^a^Intubation success rate: calculated using total randomized number of patients in group LW (n = 30), including the six patients in whom intubation failed after two attempts.

^b^Intubation success rate: calculated using total randomized number of patients in group DL (n = 30), including the one patient in whom intubation failed after two attempts.

Significant comparisons are detailed using the following footnotes:

*p<0.05

**p<0.01 compared with Group LW

^†^ *p*<0.05

^††^ *p*<0.01 compared with Group DL.

Although the intubation time was significantly longer in the WEI group (110.8±18.3 s) than the LW group (63.3±27.5 s, *p* = 0.000) or DL group (66.7±29.4 s, *p* = 0.000), the lowest SpO_2_ in WEI group was significantly higher than other two groups. The WEI JET significantly reduced successful tracheal intubation attempts compared to the LW group (1.0±0 VS. 1.21±0.4, *p* = 0.043). There was no difference in time taken for intubation and number of intubation attempts between LW group and DL group **(Table 2**).

There was no incidence of dental trauma in either group and the severity of sore throat was similar among three groups (*p* = 0.185) (**Table 2**).

## Discussion

When a difficult airway is expected or unstable cervical spine is suspected, awake flexible fiberoptic intubation with awake positioning is recommended as a safe and sure way of airway control. However, awake fiberoptic intubation usually requires patient cooperation [16]. The failure rate at the first attempt is high (approximately 53.3%), which means it requires great skills acquired from extensive experience [17]. In addition, it may be unrealistic in emergency situations due to the longer time required for intubation using awake fiberoptic approach. Considering all these shortcomings of awake fiberoptic intubation, new techniques or approaches are needed to manage the challenging tracheal intubation in patient with unstable cervical spine, especially in the emergent situation. Neurologic injury during intubation may happen because of cervical spine movement in these patients [18–19]. Lightwand was recommended for its simple but effective procedure and less linear motion compared to direct Macintosh laryngoscopy [20].

This study demonstrated that glottic visualization failed (C/L grade 3 or 4) in 90% patients with the cervical immobilization under direct laryngoscopy. The overall intubation success rate was only 80% (24/30) in the LW group, being a semi-blind technique [12]. One study including 950 patients showed that all lightwand failures were resolved with direct laryngoscopy. Similarly, all failures of direct laryngoscopy were resolved with lightwand [5]. However in this study, we have 6/30 patient failed tracheal intubation using lightwand only, compared only 1/30 in the DL group.

The epiglottis is always in contact with the posterior pharyngeal wall and consequently makes passing underneath the epiglottis is difficult for the lightwand [21]. When using direct laryngoscopy to aid lightwand, the epiglottis was lifted off the posterior pharyngeal wall and consequently leads to unhindered passage of lightwand. Biehl and Bourke revealed that the lightwand could improve the view in the hypopharynx, and transillumination could assist a blind tracheal intubation with direct laryngoscopy [22].

Despite the above mentioned advantage on combination of lightwand with direct laryngoscope, there is no approach of oxygenation and ventilation during the process of tracheal intubation. Utilizing the open feature of jet ventilation, the striking advantage of WEI JET is to provide supraglottic or infraglottic jet oxygenation and ventilation during the process of tracheal intubation under direct laryngoscopy and facilitate tracheal intubation in grade III glottis view [7,23]. This new technique obviously can prolong the period of tracheal intubation without hypoxia, minimizing hypoxia related complications. Indeed, we demonstrated that all 30 patients were successfully intubated at fist time using WEI JET and lightwand guidance under direct laryngoscopy. In comparison with other two groups, the WEI JET reduced the number of tracheal intubation attempts, significantly, with significantly increased successful rate and decreased risk of hypoxia. Although many new airway devices have been developed that can be used for difficult tracheal intubation. However, few devices can provide oxygenation and ventilation during the process of tracheal intubation. The WEI JET is a special tracheal tube which can supply oxygenation during intubation without the need of mask ventilation and assist tracheal intubation blindly. It had been demonstrated that the SaO_2_ of apnoeic pig was maintained above 95% for at least 20 min without mask ventilation in a previous animal study. In this study, breath sounds, chest rise, and monitoring of PetCO_2_ helped guide blind intubation [23]. In addition, the direct laryngoscopy could improve the view in the hypopharynx, and could assist in guiding the tip of the lightwand to pass underneath the epiglottis. This combined approach may be particularly useful when an unanticipated C/L grade 3 or 4 laryngoscopic view is encountered. It can be assumed that using both instruments together would lead to a higher success rate of tracheal intubation with reduced hypoxia-related complications, should one device alone fail. For example, the lightwand has been combined successfully with other intubating techniques including intubation through a laryngeal mask airway (LMA) [24–25].

Although the WEI JET took longer time for tracheal intubation than the lightwand in the study, but the ability to provide oxygenation/ventilation make the tracheal intubation smoother and safer. We found no difference in airway complications among all three groups.

There are still limitations in our study. First, our study is lack of a group which the WEI JET combined with direct laryngoscopy only, which means without lightwand. However, previous studies have demonstrated the advantages of WEI JET under direct laryngoscopy [23]. Second, observation bias may exist in the recordings for the unblinded design of this study. Third, all tracheal intubations were performed by an experienced anesthesiologist who is familiar with these techniques, therefore our results may not be suitable to inexperienced person.

## Conclusions

The WEI JET combined with lightwand under direct laryngoscopy can help to assist tracheal intubation and improve oxygenation during tracheal intubation in patients with difficult airway secondary to unstable cervical spine.

## Supporting Information

S1 Protocol The trial study protocol in English. (DOC)

S2 Protocol The trial study protocol in chinese. (DOC)

S1 CONSORT Checklist The CONSORT checklist. (DOC)

S1 Dataset The original data of the three groups. (XLS)

S1 Fig The CONSORT flowchart. (TIF)
