# Supplementary material for: Laryngoscope and a New Tracheal Tube Assist Lightwand Intubation in Difficult Airways due to Unstable Cervical Spine
Source: PLoS One. 2015 Mar 24;10(3):e0120231. doi: 10.1371/journal.pone.0120231 (PMC4372550; doi:10.1371/journal.pone.0120231)
Supplement: S1 Protocol — (DOC) [file pone.0120231.s001.doc]

**Project Title**:

# Laryngoscope and a new tracheal tube assists lightwand intubation in difficult airway

**Principal Investigator**:

Wuhua Ma, M.D., PhD

Department of Anesthesiology

The First Affiliated Hospital of Guangzhou University of Traditional Chinese Medicine

16 Jichang Road, Guangzhou, 510405, China

E-mail: [yingcao1986@163.com](mailto:yingcao1986@163.com)

Phone number: +8613580315308

Fax number: +8602036591230

**Sub - Investigators:**

Huafeng Wei, M.D., PhD. Assistant Professor Anesthesia;Caineng Wu, M.D.

Mar 1, 2014

**Research Plan**

**Hypothesis**

Tracheal intubation is frequently required in patients with acute respiratory failure and in almost all patients under pulmonary or cardiac resuscitation. However, difﬁcult tracheal intubation may occurs in 1-4% of all types of patients, and even range between 6% and 11% in emergency department[1-2]. Adverse outcomes associated with difﬁcult airway management reported in the Closed Claims Analysis from the Committee on Professional Liability of the American Society of Anesthesiologists constitute the largest class of injury, with death and brain injury occurring in 85% of those case[3-4]. The major cause of the intubation-related brain damage or death, especially in emergency situations, is a lack of adequate oxygenation and ventilation with conventional mechanical positive pressure ventilation through an open airway during tracheal intubation.

Patients with cervical spine instability and limited range of motion are challenge to anesthesiologists. The anesthesiologist must consider two potentially competing objectives, that is, securing the airway while minimizing neck motion. Manual in-line stabilization (MILS) is recommended during direct laryngoscopy and intubation in patients with known or suspected cervical spine instability[5]. However, MILS often worsens direct laryngeal visualization and therefore gives cause for difficult tracheal intubation [6].

The lightwand is a simple technique that has been proven to be safe and effective in the cases of difﬁcult intubation[7-9]. The tracheas of patients with unstable cervical spines could not be safely managed by direct laryngoscopy. It was recommend the use of the lightwand in the setting of a presumed unstable cervical spine injury over the Macintosh laryngoscope[10]. A special tracheal tube designed by Wei named WEI JET (WEI JET; Wei Medical LLC, Cherry Hill, NJ) which combined with supraglottic jet ventilation (SJV) supplies oxygenation and ventilation during tracheal intubation and assists tracheal intubation blindly in patients with difficult airway[11].

**Aim**

Testing the efficacy and safety of direct laryngoscopy and WEI JET combined with lightwand would assist tracheal intubation, especially in patients with difficult airway.

**Summary of the WEI JET**

The WEI JET is a special tracheal tube designed by Wei (WEI JET; Wei Medical LLC, Cherry Hill, NJ) which combined with supraglottic jet ventilation (SJV) supplies oxygenation and ventilation during tracheal intubation and assists tracheal intubation blindly in patients with difficult airway[11]. The supraglottic jet oxygenation and ventilation is a model of non-invasive jet oxygenation/ventilation, which has been shown to provide effective oxygenation and ventilation in an animal model and clinical patients with minimal complications [12-14]. Previous studies have demonstrated that supraglottic jet ventilation (SJV) effectively maintained oxygenation and ventilation during difficult intubation, with minimal complication[13-14]. One of the major concerns using jet ventilation is the serious complication of barotraumas, which usually occurred by injecting high pressure gases into a semi or fully closed tissue cavity (e.g. transtracheal jet ventilation in patients with significant upper airway obstruction) [14-15], which may occur as high as 10% during the use of emergent transtracheal jet ventilation [16]. We tend to minimize the risks of barotraumas in this study attributing to the followings:The safe working parameters (driving pressure 15 psi, respiratory rate 20/min, and I/E ratio 1:2) for SJV, can reduce the incidence of barotrauma (subcutaneous emphysema, pneumothorax, stomach insufflation etc.)

**Summary of preliminary studies**

The WEI JET is a special tracheal tube which can supply oxygenation using SJV during intubation without the need of mask ventilation and assist tracheal intubation blindly. It had been revealed that the SpO_2_ of apnoeic pig was maintained above 95% for at least 20 min without mask ventilation in our previous animal study. In this study, breath sounds, chest rise, and monitoring of PetCO_2_ helped guide blind intubation[12].

This study provide the scientific basis and background for our research plan.

**Study Design**

This will be a prospective randomized clinical trial.

**Study Objectives**

The purpose of the study is to test the efficacy and safety of direct laryngoscopy and WEI JET combined with lightwand would assist tracheal intubation, especially in patients with difficult airway.

**Duration**

It is anticipated that it will take about six months to complete this study. The study plans to begin in March 2014 and will plan to be completed in September 2014.

**Subject Recruitment and Selection**

Ninety patients with unstable cervical spine disorders (ASA Ⅰ～Ⅲ) for general anaesthesia were enrolled. The patients recruited in this study were randomly by computer-generated randomization schedule into the LW group (intubations were performed only using lightwand), the DL group (lightwand used in conjunction with direct laryngoscopy for intubation), and the WEI group (the WEI JET take the place of regular tracheal tube which combined with the direct laryngoscopy and lightwand). The intubation time, number of intubation attempts and success rate of intubation were monitored.

**Inclusion Criteria**

Ninety patients with unstable cervical spine disorders (ASA Ⅰ～Ⅲ) for general anaesthesia were enrolled.

**Exclusion Criteria**

Age <18 years; had risk factors for gastric aspiration; increased intracranial pressure; and obesity (BMI> 35); relevant drug allergy; abnormalities of the upper airway, polyps, tumours, abscesses, inflammation, or foreign bodies in the upper airway.

**Obtaining the institutional approval, patient consents**

The ethical approval was obtained from the Ethics Committee of the First Affiliated Hospital of Guangzhou University of Traditional Chinese Medicine and written informed consent was obtained from all the patients.

**Experimental group**

These patients were randomized by computer-generated randomization schedule into one of three groups, with 30 patients in each group: the LW group, the DL group

and the WEI group.

The LW group:

Tracheal intubations were performed only using lightwand in which introduced a regular tracheal tube (7-mm ID tube in women, and an 7.5-mm ID tube in men), and the tip of the tube/lightwand combination was bent to a 90° angle[17]. The room lights were dimmed, and the combination was introduced into the oral cavity and repositioned in the midline until its entry into the oropharynx. When the tip of lightwand was placed inside the glottis, a well-defined circumscribed glow could be seen in the anterior neck[18]. If oesophageal intubation happened, the transmitted glow was diffuse and the procedure was tried again. After removal of the lightwand, proper tracheal tube placement was confirmed by end-tidal carbon dioxide monitoring.

The DL group:

Use of lightwand in combination with direct laryngoscopy for tracheal intubation. A Macintosh size 3 blade was used for lightwand intubations. For all intubations with this device, the blade was held in the non-dominant hand prior to insertion of the tracheal tube-lightwand combination. Tracheal intubation under direct vision of the glottis (C/L Grade 1 or 2). For patients with Grade 3 glottis view, the lightwand was passed underneath the epiglottis. For Grade 4, tracheal intubation with midline technique[19]. When an optimal transillumination was obtained, tracheal intubation was achieved. If transillumination was not seen in larynx, the procedure was tried again.

The WEI group:

The WEI JET took the place of regular tracheal tube which combined with the direct laryngoscopy and lightwand. As shown in the Figure 1, The WEI JET consisted of two parts: an additional catheter for end-tidal CO_2_ pressure (PetCO_2_) monitoring and a jet catheter for ventilation. The jet catheter with an ID 2.0 mm which was built into the anterior wall of WEI JET. Ventilation using a manual jet ventilator (Manujet Ⅲ, VBM Medizintechnik GmbH, Germany) connected to the end of jet catheter. We readjusted the distal tip of the lightwand until midline illumination was observed in the anterior neck , the maximum stable PetCO_2_ and chest rise were achieved[11]. The WEI JET was slid into the trachea.

**Anesthesia induction**

Each patient was placed in supine position and the head in a neutral position. MILS was created with a cervical immobilization collar (Stifneck Select, Leardal Medical GmbH, Germany). They were given 5 min of pre-oxygenation before anesthesia induction. Anaesthesia was induced with propofol target control infusion (4μg.ml^-1^) and remifentanil (3.5ng.ml^-1^) followed by succinylcholine (1mg.kg^-1^).

**Monitoring and observation**

Non-invasive blood pressure, SpO_2_, ECG were monitored and recorded every minute. The glottis view was described by Cormack and Lehane (C/L) and divided into four grades (1=full view of the glottis; 2=partial view of the glottis; 3=only the epiglottis visible; and 4=neither epiglottis nor glottis visible.), without applying external laryngeal pressure.

The primary endpoints were the time taken to intubate and C/L grades. The intubation time was deﬁned as from insertion of the intubation device between the teeth to the ﬁrst breath of controlled mechanical ventilation and detection of positive PetCO2. The secondary endpoints were the number of intubation attempts and success rate of intubation. After the procedure, patients were asked about the severity of sore throat using a VAS (0 = no pain, 10 = worst imaginable pain) at discharge from the post-anesthesia recovery room.

**Statistical analysis**

All data were reported as mean ± SD, absolute number (n), or percentages. Statistical analyses were performed with the Statistical Package for Social Sciences Software (SPSS 18.0 for windows; SPSS Inc., Chicago, IL, USA). The intubation time, lowest SpO_2_ during intubation, VAS score and number of intubation attempts among the three groups were analysed by ANOVA and post hoc tests. Data for the rate of successful intubation were analysed using a chi-squared test. All values A P-value < 0.05 was considered to be statistically significant

**Confidentiality**

This study will collect Personal Health Information. Upon enrollment, name, date of birth, and medical record number will be recorded on a subject data form, as well as admitting diagnosis and a summary of the patient’s past medical history and current medication list.

**Potential Risks**

Risks of intubation are small but nonetheless possible. We have taken several measures to protect life safety by the surgical and anesthesia team.

**References**

1. Orebaugh SL. Difﬁcult airway management in the emergency department. J Emerg Med 2002;22:31—48.
2. Combes X, Le Roux B, Suen P, et al. Unanticipated difﬁcult airway in anesthetized patients: prospective validation of a management algorithm. Anesthesiology 2004;100:1146—50.
3. Caplan RA, Vistica MF, Posner KL, Cheney FW. Adverse anesthetic outcomes arising from gas delivery equipment: a closed claims analysis. Anesthesiology 1997;87:741—8.
4. Schwartz DE, Matthay MA, Cohen NH. Death and other complications of emergency airway management in critically ill adults. A prospective investigation of 297 tracheal intubations. Anesthesiology 1995;82:367—76.
5. Huang WT, Huang CY, Chung YT. Clinical comparisons between GlideScope video laryngoscope and Trachlight in simulated cervical spine instability. J Clin Anesth 2007; 19: 110-4.
6. Heath KJ. The effect of laryngoscopy of different cervical spine immobilization techniques. Anaesthesia 1994; 49: 843-5.
7. Ovassapian A, Meyer RM. Airway Management. In: Longnecker JH,Tinker JH,MorganGE, eds. Principles and Practice of Anesthesiology, 2nd edn. St. Louis:Mosby, 1998: 1064-99.
8. Hung OR, Stewart RD. Illuminating stylet (lightwand). In: Benumof JL, ed. Airway Management,St. Louis: Mosby, 1996: 342-52.
9. Hung OR, Pytka S, Morris I, et al. Clinical trial of a new lightwand device (Trachlight) to intubate the trachea. Anesthesiology 1995; 83: 509-14.
10. Prasarn ML, Conrad B, Rubery PT, et al. Comparison of 4 airway devices on cervical spine alignment in a cadaver model with global ligamentous instability at C5-C6. Spine 2012; 37: 476-81.
11. Peng J, Ye J, Zhao Y ,et al. Supraglottic jet ventilation in difficult airway management.J Emerg Med 2012; 43: 382-90.
12. Wei HF. A new tracheal tube and methods to facilitate ventilation and placement in emergency airway management. Resuscitation 2006; 70:438-44.
13. Dziewit JA, Wei H. Supraglottic Jet Ventilation Assists Intubation in a Marfan’s Syndrome Patient with a Difficult Airway. Journal of Clinical Anesthesia 2011; 23(5), 407–09.
14. Dziewit JA, Wei H. Supraglottic Jet Ventilation Assists Intubation in a Patient with Difficult Airway Due to Unrecognizable Supraglottic Structures. J Anesthe Clinic Res 2011, 2(6):141.
15. hra G, Gockner G, Kashanipour A, Aloy A: High-frequency jet ventilation in European and North American institutions: developments and clinical practice. European Journal of Anaesthesiology 2000;17(7):418-30.
16. Benumof JL, Scheller MS. The importance of transtracheal jet ventilation in the management of the difficult airway. Anesthesiology 1989; 71(5): 769-778.
17. Chen J, Lou W,Wang E,Lu K. Optimal bent length of lightwand for intubation in adults: a randomized, prospective, comparative study. J Int Med Res 2012; 40: 1519-31.
18. Agrò F,Hung OR,Cataldo R,Carassiti M,Gherardi S. Lightwand intubation using the Trachligh: a brief review of current knowledge. Can J Anaesth 2001; 48: 592-9.
19. Agro F, Benumof JL, Carassiti M, Cataldo R, Gherardi S, Barzoi G. Efficacy of a combined technique using the Trachlight together with direct laryngoscopy under simulated difficult airway conditions in 350 anesthetized patients. Can J Anaesth 2002; 49: 525-6.
