# Supplementary material for: Laryngoscope and a New Tracheal Tube Assist Lightwand Intubation in Difficult Airways due to Unstable Cervical Spine
Source: PLoS One. 2015 Mar 24;10(3):e0120231. doi: 10.1371/journal.pone.0120231 (PMC4372550; doi:10.1371/journal.pone.0120231)
Supplement: S2 Protocol — (DOC) [file pone.0120231.s002.doc]

**直接喉镜和魏氏喷气导管辅助光棒在处理困难气道的应用**试验方案

项目名称：**直接喉镜和魏氏喷气导管辅助光棒在处理困难气道的应用**

计划类别：临床研究

承担单位：广州中医药大学

推荐单位：广州中医药大学

项目负责人：马武华 手机：13318860680

项目联系人：吴财能 手机：13580315308

电子邮箱：[yingcao1986@163.com](mailto:yingcao1986@163.com)

申请日期：2014 年3月1日

1. 研究题目：

**直接喉镜和魏氏喷气导管辅助光棒在处理困难气道的应用**

研究背景：

许多患者接受全身麻醉何心肺复苏时需要气管插管和常规机械通气。但是，困难气管插管发生率可达1%-4%，特别是在急诊科高达6%-11%[1-2]。美国ASA报道。由于困难气道处理失败占所有因素导致死亡和大脑损伤的85%[3-4]。与气管插管相关的脑损伤与死亡的主要原因是，在气管插管过程中缺乏一个通道来正压通气提供足够的氧合和通气。

有潜在不稳定的颈椎病患者的气道管理是一个复杂而艰巨的任务。麻醉医师必须同时处理两个重要和相互矛盾的问题，即尽量减少颈椎运动度，以防止造成和加重神经损伤；和快速、高效的建立气道。MILS（manual in-line stabilization）是颈椎不稳定的患者经口气管插管推荐使用的方法[5]。但是，MILS往往会阻碍直接喉镜暴露声门，从而导致气管插管困难[6]。

光棒是使用透照的原理进行气管插管，已被证明是一种简单和有效的困难气道处理技术[7-9]。颈椎病不稳定患者用直接喉镜来气管插管不是一个安全的选择。在预计有不稳定颈椎损伤患者的气管插管中光棒比直接喉镜更值得推荐使用[10]。

魏氏喷射气管导管是美籍华人魏华峰教授发明的新型气管导管。魏氏喷射气管导管结合声门上喷射通气可以为呼吸暂停患者气管插管的时提供充足的氧气和通气，并有助于促进气管插管成功[11]。

1. 目标：

评价直接喉镜和魏氏喷射气管辅助光棒处理困难气道的安全性和稳定性，为困难气道的处理提供新方法。

1. WEI JET的介绍：

魏氏喷射气管导管是美籍华人魏华峰教授发明的新型气管导管。魏氏喷射气管导管结合声门上喷射通气可以为呼吸暂停患者气管插管的时提供充足的氧气和通气，并有助于促进盲探气管插管成功[11]。声门上喷射通气装置可以为动物模型和临床上的病人提供有效的通气和氧供，并且少有并发症[12-14]。我们的前期研究显示，在困难气管插管时，声门上喷射通气装置能提供有效的氧供和通气且少有并发症[13-14]。有关声门上喷射通气装置并发症的一个主要的担忧就是气压伤。它的发生主要原因是，在一个相对或完全密闭的组织腔隙内注入高压的气体，例如对严重声门上气道梗阻的病人使用经气管声门上喷射通气[14-15]。这种紧急经气管声门上喷射通气发生气压伤的几率高达10%[16]。本次研究中为了防止气压伤的发生，须符合以下原则：声门上喷射通气时使用安全的工作参数。例如驱动压力：15 psi，通气频率 20/min，吸/呼 1：2。以防止皮下气肿，气胸和胃内压增高等。

1. 有关WEIJET的前期研究：

WEI JET是一种特殊的气管导管，它可以同声门上喷射通气装置结合，为呼吸暂停患者气管插管的时提供充足的氧气和通气而无需面罩通气，并有助于促进盲探气管插管成功。我们的前期动物实验显示，WEI JET同声门上喷射通气装置结合用于窒息的猪，可以维持20分钟使SpO_2_在95%以上而无需面罩通气。在这个研究中证实，呼吸音，胸廓抬起和呼吸末二氧化碳检测可以有助于促进盲探气管插管成功[12]。

这为我们的研究提供了扎实的基础。

1. 实验设计：

本临床研究是随机对照实验设计。

1. 研究目的：

本研究的主要目的是，评价直接喉镜和魏氏喷射气管辅助光棒处理困难气道的安全性和稳定性，为困难气道的处理提供新方法。

1. 研究期限：

本研究预计需要为期半年的时间完成（2014年3月至2014年9月）。

1. 受试者招募：

经我院医学伦理委员会批准，选择颈椎不稳定病需全麻气管插管手术的患者90名（ASA Ⅰ～Ⅲ）。招募入组后采用电脑随机生成分配入以下三组：LW组（仅用光棒完成气管插管）、DL组（光棒符合直接喉镜完成气管插管）和WET 组（WEI JET代替普通气管导管结合光棒和直接喉镜来完成气管插管）。插管时间、插管次数和插管成功率等指标被记录。

纳入和剔除标准：

纳入：椎不稳定病需全麻气管插管手术的患者90名（ASA Ⅰ～Ⅲ）被纳入。剔除：年龄下于18岁；胃反流危险因数者；有相关药物过敏；颅内高压；BMI> 35；上呼吸道异常（息肉，肿物，炎症，息肉等）。

1. 知情同意：

本研究申请获得广州中医药大学第一附属医院伦理委员会的批准，并同以的患者签署同意书。

1. 实验分组：

颈椎不稳定病需全麻气管管手术的患者90名。招募入组后采用电脑随机生成分配入以下三组：LW组、DL组和WET 组，每组各30例病人。

LW组：仅用光棒完成气管插管。光棒套入普通气管导管（女性7.0ID，男性7.5ID），并将光棒和气管导管的复合体的前端弯曲成 90°的角[17]。调暗手术室的灯光，将光棒-导管复合体置入患者的口角，当进入口咽后复位正中线。当光棒前端靠近声门时，在患者的颈前区的位置可以看到清晰局限的光斑[18]。如果误入食道，由光棒投射的光斑是弥散的，重复上诉步骤。移除光棒后，导管在气管内通过呼吸末二氧化碳监测来证实。

DL组：通过光棒符合直接喉镜完成气管插管。3号的Macintosh喉镜被用来辅助光棒气管插管。在置入光棒前用非优势手握3号的Macintosh喉镜辅助气管插管。当C/L 分级是 1 or 2 时，则直接气管插管；当C/L 分级是 3时，光棒通过会厌下面完成气管插管；当C/L 分级是 4，采用中线技术完成气管插管[19]。当颈前区的位置可以看到清晰局限的光斑时，气管插管就成功了，反之重复上诉步骤。

WET 组：WET JET 代替普通气管导管来结合直接喉镜和光棒。如Figure 1所示，WEI JET 由两部分组成：一个额外的导管监测呼吸末二氧化碳和一个内至导管来做喷射通气。来于喷射通气的导管至于WEI JET导管壁内（2.0mm）。我们调整光棒前端，当患者的颈前区的中间的位置看到清晰局限的光斑时，手控喷射通气装置，同时见胸阔抬起和高大的呼吸末二氧化碳波形时，将导管至入气管内[11]。

1. 麻醉诱导：

每个病人入室后仰卧位，头部通过颈椎固定圈(Stifneck Select, Leardal Medical GmbH, Germany)来实现MILS体位。诱导前面罩吸氧5分钟。麻醉诱导，TCI丙泊酚 (4μg.ml^-1^)和瑞米芬太尼 (3.5ng.ml^-1^)后给予司可林(1mg.kg^-1^)。

1. 监测与观察：

每分钟无创监测BP，SpO_2_， ECG 。声门暴露分级通过Cormack and Lehane (C/L)分为四级（1=声门全部被显露，2=声门部分被显露，3=仅显露会厌，4=会厌或声门都无法显露）。

主要观察指标：插管时间、C/L分级。插管时间被定义为，从插管设备接触门齿到控制呼吸见阳性上午二氧化碳波形。插管次数和插管成功率。当病人离开麻醉恢复室前，喉咙痛疼VAS评分被观察。

1. 统计学处理：

所得全部数据采用SPSS17.0统计软件进行统计学处理。计量资料以均数±标准差（$\bar{x}\pm s$）表示，组间比较采用t检验；不同时点比较采用重复测量数据方差分析。计数资料比较采用χ^2^检验。P＜0.05为差异有统计学意义。

**References**

1. Orebaugh SL. Difﬁcult airway management in the emergency department. J Emerg Med 2002;22:31—48.
2. Combes X, Le Roux B, Suen P, et al. Unanticipated difﬁcult airway in anesthetized patients: prospective validation of a management algorithm. Anesthesiology 2004;100:1146—50.
3. Caplan RA, Vistica MF, Posner KL, Cheney FW. Adverse anesthetic outcomes arising from gas delivery equipment: a closed claims analysis. Anesthesiology 1997;87:741—8.
4. Schwartz DE, Matthay MA, Cohen NH. Death and other complications of emergency airway management in critically ill adults. A prospective investigation of 297 tracheal intubations. Anesthesiology 1995;82:367—76.
5. Huang WT, Huang CY, Chung YT. Clinical comparisons between GlideScope video laryngoscope and Trachlight in simulated cervical spine instability. J Clin Anesth 2007; 19: 110-4.
6. Heath KJ. The effect of laryngoscopy of different cervical spine immobilization techniques. Anaesthesia 1994; 49: 843-5.
7. Ovassapian A, Meyer RM. Airway Management. In: Longnecker JH,Tinker JH,MorganGE, eds. Principles and Practice of Anesthesiology, 2nd edn. St. Louis:Mosby, 1998: 1064-99.
8. Hung OR, Stewart RD. Illuminating stylet (lightwand). In: Benumof JL, ed. Airway Management,St. Louis: Mosby, 1996: 342-52.
9. Hung OR, Pytka S, Morris I, et al. Clinical trial of a new lightwand device (Trachlight) to intubate the trachea. Anesthesiology 1995; 83: 509-14.
10. Prasarn ML, Conrad B, Rubery PT, et al. Comparison of 4 airway devices on cervical spine alignment in a cadaver model with global ligamentous instability at C5-C6. Spine 2012; 37: 476-81.
11. Peng J, Ye J, Zhao Y ,et al. Supraglottic jet ventilation in difficult airway management.J Emerg Med 2012; 43: 382-90.
12. Wei HF. A new tracheal tube and methods to facilitate ventilation and placement in emergency airway management. Resuscitation 2006; 70:438-44.
13. Dziewit JA, Wei H. Supraglottic Jet Ventilation Assists Intubation in a Marfan’s Syndrome Patient with a Difficult Airway. Journal of Clinical Anesthesia 2011; 23(5), 407–09.
14. Dziewit JA, Wei H. Supraglottic Jet Ventilation Assists Intubation in a Patient with Difficult Airway Due to Unrecognizable Supraglottic Structures. J Anesthe Clinic Res 2011, 2(6):141.
15. hra G, Gockner G, Kashanipour A, Aloy A: High-frequency jet ventilation in European and North American institutions: developments and clinical practice. European Journal of Anaesthesiology 2000;17(7):418-30.
16. Benumof JL, Scheller MS. The importance of transtracheal jet ventilation in the management of the difficult airway. Anesthesiology 1989; 71(5): 769-778.
17. Chen J, Lou W,Wang E,Lu K. Optimal bent length of lightwand for intubation in adults: a randomized, prospective, comparative study. J Int Med Res 2012; 40: 1519-31.
18. Agrò F,Hung OR,Cataldo R,Carassiti M,Gherardi S. Lightwand intubation using the Trachligh: a brief review of current knowledge. Can J Anaesth 2001; 48: 592-9.
19. Agro F, Benumof JL, Carassiti M, Cataldo R, Gherardi S, Barzoi G. Efficacy of a combined technique using the Trachlight together with direct laryngoscopy under simulated difficult airway conditions in 350 anesthetized patients. Can J Anaesth 2002; 49: 525-6.
