# Supplementary figures and images for: Laryngoscope and a New Tracheal Tube Assist Lightwand Intubation in Difficult Airways due to Unstable Cervical Spine
Source: PLoS One. 2015 Mar 24;10(3):e0120231. doi: 10.1371/journal.pone.0120231 (PMC4372550; doi:10.1371/journal.pone.0120231)

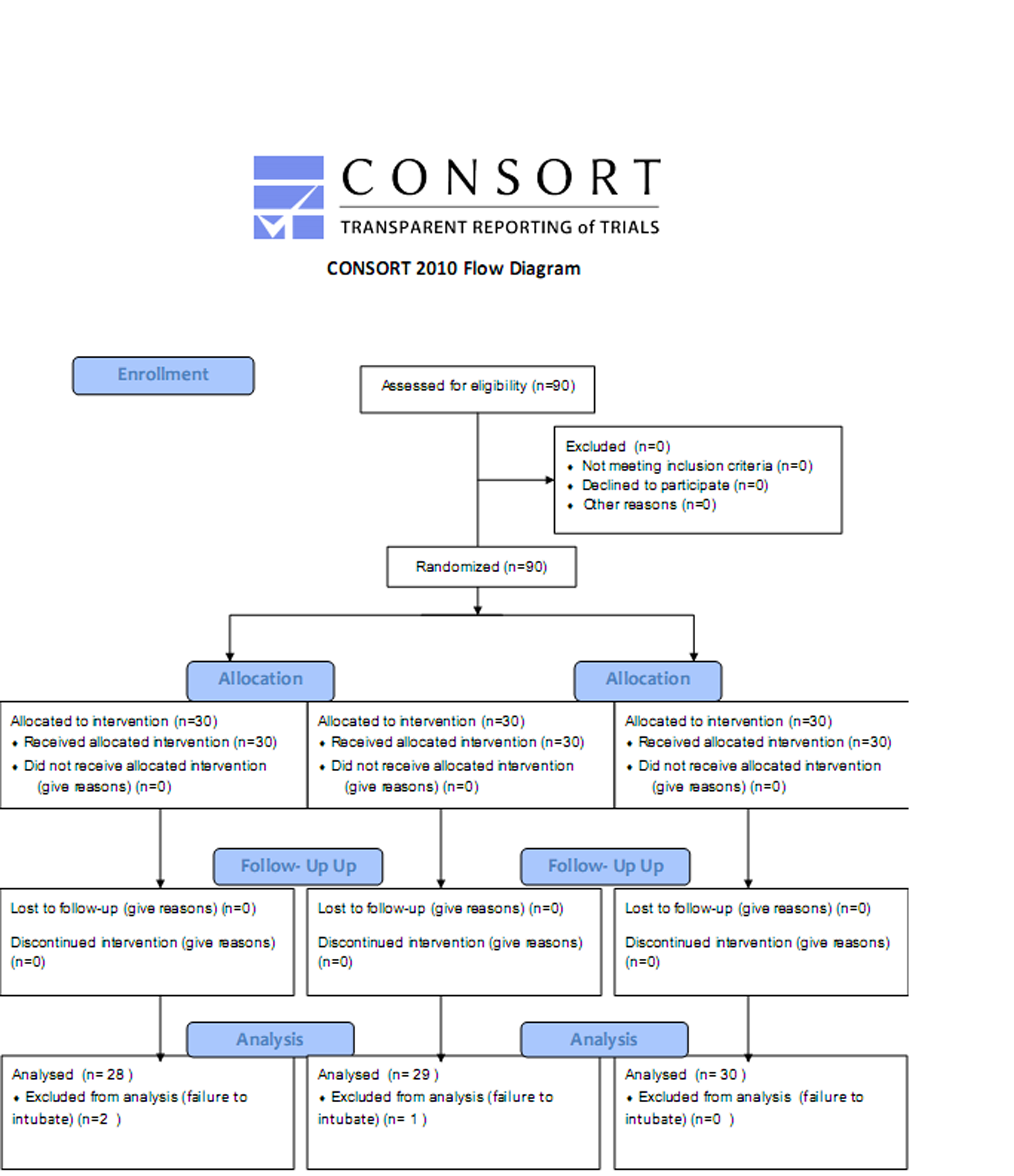

Supplement: S1 Fig — (TIF) [file pone.0120231.s005.tif]
